# Spatial-Temporal Evolution of PM_2.5_ Concentration and its Socioeconomic Influence Factors in Chinese Cities in 2014–2017

**DOI:** 10.3390/ijerph16060985

**Published:** 2019-03-19

**Authors:** Yazhu Wang, Xuejun Duan, Lei Wang

**Affiliations:** 1 Nanjing Institute of Geography and Limnology, Chinese Academy of Sciences, Nanjing 210008, China; wangyazhu0428@163.com (Y.W.); wanglei@niglas.ac.cn (L.W.); 2 College of Resources and Environment, University of Chinese Academy of Sciences, Beijing 100049, China; 3 Key Laboratory of Watershed Geographic Sciences, Nanjing Institute of Geography and Limnology, Chinese Academy of Sciences, Nanjing 210008, China

**Keywords:** PM_2.5_ concentration, spatial-temporal evolution, socioeconomic influence factors, China

## Abstract

PM_2.5_ is a main source of China’s frequent air pollution. Using real-time monitoring of PM_2.5_ data in 338 Chinese cities during 2014–2017, this study employed multi-temporal and multi-spatial scale statistical analysis to reveal the temporal and spatial characteristics of PM_2.5_ patterns and a spatial econometric model to quantify the socio-economic driving factors of PM_2.5_ concentration changes. The results are as follows: (1) The annual average value of PM_2.5_ concentration decreased year by year and the monthly average showed a U-shaped curve from January to December. The daily mean value of PM_2.5_ concentration had the characteristics of pulse-type fluctuation and the hourly variation presented a bimodal curve. (2) During 2014–2017, the overall PM_2.5_ pollution reduced significantly, but that of more than two-thirds of cities still exceeded the standard value (35 μg/m^3^) regulated by Chinese government. PM_2.5_ pollution patterns showed high values in central and eastern Chinese cities and low values in peripheral areas, with the distinction evident along the same line that delineates China’s uneven population distribution. (3) Population agglomeration, industrial development, foreign investment, transportation, and pollution emissions contributed to the increase of PM_2.5_ concentration. Urban population density contributed most significantly while economic development and technological progress reduced PM_2.5_ concentration. The results also suggest that China in general remains a “pollution shelter” for foreign-funded enterprises.

## 1. Introduction

China’s rapid industrialization and urbanization alongside high annual emissions of pollutants [1] has caused the air quality of Chinese cities to deteriorate significantly, threatening public health and urban residents’ well-being. In recent years, the frequent occurrence of haze pollution in Beijing and other megacities in China has led to a public outcry and has attracted considerable global attention [2]. Since 2013, there has been large-scale and long-term air pollution incidents in China, especially fine particulate matter of less than 2.5 microns (PM_2.5_) pollution [3]. In 2015, 265 of the 338 cities in China exceeded the PM_2.5_ standard of the World Health Organization (WHO) (10 μg/m^3^). PM_2.5_ was the most polluting component in the number of polluted days, accounting for 66.8% of the exceeded days. As PM_2.5_ refers to fine particles with a small aerodynamic equivalent diameter, it is the most important characteristic pollutant of atmospheric composite pollution [4,5]. Research has shown that PM_2.5_ can damage human lung tissue, aggravate chronic respiratory and cardiovascular diseases, and increase the risk of cancer in exposed populations [6,7]. The risk of emergency hospitalization for cardiovascular and cerebrovascular diseases increased by 1.29% per 10 μg/m^3^ increase in the concentration of PM_2.5_. In 2013, the International Agency for Research on Cancer listed PM_2.5_ as a human carcinogen [8]. In addition, PM_2.5_ can be transmitted over a long distance, stay in the atmosphere for a long time, reduce atmospheric visibility, adversely affect transportation, delay flights or lead to their cancellation, and cause highway closures [9]. High PM_2.5_ pollution has even forced schools to suspend classes in primary and secondary schools [10].

The serious harm resulting from PM_2.5_ has led the academic community to pay close attention to the issue and carry out large-scale PM_2.5_ research. The research mainly focuses on the features of PM_2.5_ pollution [11], its chemical composition, source analysis, health effects [12], cross-border pollution [13], and the impact of PM_2.5_ on atmospheric visibility [14] and human health. Most of the studies are based on pilot experiment monitoring results for a given city or region. The data range is small and the time scale is short. The spatial estimation and spatial characteristic analysis of PM_2.5_ concentration mainly use the following four data types and methods to estimate PM_2.5_ concentration: Remote sensing image to retrieve atmospheric aerosol thickness [15], real-time data spatial interpolation of the monitoring points [16], a regression model [17], and a hybrid model [18]. PM_2.5_ research has gradually shifted from small scale to medium scale and large scale. The research content has also changed from models evaluating PM_2.5_ concentration to those investigating the influence mechanism. The influencing factors of PM_2.5_ mainly focus on climatic meteorological conditions and social and economic activities, which all play an important role in the formation and diffusion of PM_2.5_. In terms of temporal and spatial distribution characteristics of PM_2.5_ concentration, the research mainly focuses on different time scales of a certain region or country, revealing the evolution of its temporal and spatial patterns, and looking for scientific and effective pollution control methods. In terms of climate and meteorology factors, urban PM_2.5_ concentration is related to temperature, precipitation, wind speed, atmospheric pressure, atmospheric humidity, total radiation, and so on. In terms of social and economic factors, urban PM_2.5_ concentration has certain correlation with population agglomeration [19], economic growth, industrial structure, fossil energy consumption [20], and traffic conditions. In recent years, several studies have been published on the factors that promote socio-economic pollution in China. The studies found that population concentration, economic development, inter-regional trade, industrialization, urbanization, vehicle emissions, city size, and energy use exacerbated PM_2.5_ pollution [21].

To cope with the frequent occurrence of haze pollution, China’s Ministry of Environmental Protection newly incorporated PM_2.5_ into environmental air quality monitoring, via the Environmental Air Quality Standard (GB 3095-2012), and set corresponding standards. Thereafter, the Ministry of Environmental Protection developed a monitoring implementation plan, set up automatic monitoring points for air quality, and published PM_2.5_ real-time monitoring data for the public. A total of 945 monitoring points were set up in 190 cities in 2014, 2015, and 2016, and the monitoring scope was expanded to 1436 monitoring points in 338 cities in 2017. In addition, in response to serious and persistent PM_2.5_ pollution, the State Council of China announced its goal of reducing PM_2.5_ concentration by 25% from 2012 to 2017. To achieve this ambitious goal, scientific research was undertaken on the temporal and spatial evolution characteristics of PM_2.5_, through quantitative research on human factors, including economic growth, population agglomeration, urbanization, and industrialization. The research concluded that it was especially important for Chinese policymakers to make policies that control air pollution and make trade-offs between development and protection [21].

This study used geostatistical analysis and exploratory spatial data analysis to analyze real-time monitoring data of PM_2.5_ concentration in 2014–2017. It analyzes the pollution characteristics and temporal and spatial patterns of PM_2.5_ pollution in 329 prefecture-level cities in China from two dimensions, time and space. Moreover, this study analyzes the important factors affecting the distribution of PM_2.5_ concentration, from the perspective of social economy, in order to provide a scientific basis for air pollution control.

## 2. Materials and Methods 

### 2.1. Data Sources

The study area was mainland China (excluding Hong Kong, Macau, and Taiwan, owing to data availability restrictions). The study data were PM_2.5_ concentration monitoring values of 1436 monitoring points in 338 cities in China from 2014 to 2017. The research data were from real-time monitoring data released by the China National Environmental Monitoring Center (http://www.cnemc.cn) and the China Air Quality Online Monitoring and Analysis Platform (https://www.aqistudy.cn/historydata/index.php). The monitoring site used Thermo Fisher 1405F (Thermo Fisher Scientific, Waltham, MA, USA) to observe the concentration of PM_2.5_. The goal of this method was to cut PM_2.5_ in ambient air at a constant flow rate and to use a filter membrane dynamic measurement system and micro-oscillation balance to measure PM_2.5_ concentration [22].

According to China’s ambient air quality standards (GB 3095-2012), Chinese cities are regarded as second-class environmental functional areas, including residential areas, mixed commercial and commercial areas, industrial areas, cultural areas, and rural areas. The annual and daily limits of PM_2.5_ concentration are 35 μg/m^3^ and 75 μg/m^3^, respectively. The Environmental Air Quality Index AQI Technical Regulations (HJ633-2012), issued by the Ministry of Environmental Protection, defines pollution based on a 24 h classification. PM_2.5_ concentration at 0–35 μg/m^3^ is excellent, 35–75 μg/m^3^ is good, 75–115 μg/m^3^ is light pollution, 115–150 μg/m^3^ is moderate pollution, 150–250 μg/m^3^ is heavy pollution, and 250–500 μg/m^3^ is serious pollution.

“Daily average” refers to the arithmetic mean of a 24 h monitoring value on a natural day. “Monthly average” refers to the arithmetic mean of the average value of each day in a month. “Quarter average” refers to the arithmetic mean of the average value of each day in a quarter. “Annual average” refers to the arithmetic average of the daily averages over the course of a year. Spring is from March to May, summer is from June to August, fall is from September to November, and winter is from December to February.

In this study, socio-economic development data affecting PM_2.5_ concentration were mainly from the China Urban Statistical Yearbook (2014–2017). For some missing data, the statistical yearbooks of each province and city are referenced.

### 2.2. Methods

#### 2.2.1. Hot Spot Analysis (Getis–Ord Gi)

Hot spot analysis uses the local G statistic proposed by Getis and Ord. The calculation formula is the following:

(1) $$G_{i}^{*}=\frac{\sum_{j}w_{ij}x_{j}{\bar{x}}^{*}\sum_{j}w_{ij}}{S^{*}\sqrt{\left\{n\sum_{j}w_{ij}^{2}-{\left(\sum_{j}w_{ij}\right)}^{2}\right\}/\left(n-1\right)}}$$

In the formula, $\bar{x}$ = $\underset{j}{\sum }x_{j}/n,s^{*}=\sqrt{\underset{j}{\sum }x_{j}^{2}-{\bar{x}}_{j}^{2}}$. the G-statistic is a significant statistic. A high G-value indicates a high value spatial agglomeration, that is, a hot spot; a low G-value indicates a low value spatial agglomeration, that is, a cold spot; and a close value of 0 indicates no significant spatial correlation.

#### 2.2.2. Kriging Interpolation

Limited by physical and economic conditions, China’s environmental monitoring points are unevenly distributed, but PM_2.5_ concentration has a significant spatial autocorrelation. Interpolation accuracy of monitoring data at the regional level is more accurate than the data acquired from remote sensing inversion [23]. Therefore, the complete spatial distribution of the region can be explained by interpolation.

The Kriging interpolation method, also known as the optimal spatial covariance interpolation method, is an optimal interpolation method for spatial interpolation. It can obtain good continuity, high accuracy, and good volatility, and the results have no bias and minimum variance, which can accurately simulate the spatial distribution characteristics of PM_2.5_ [24]. The estimated value $Z^{*}\left(V\right)$ of any one of the parcels is represented by the weighted average of the observations of the adjacent parcels is the following:

(2) $$Z^{*}\left(V\right)=\overset{n}{\underset{i=1}{\sum }}\lambda_{i}Z\left(x_{i}\right)$$

In the formula, *n* is the number of adjacent samples $Z\left(x_{i}\right)$ and $\lambda_{i}$ is the weight of the observed sample $Z\left(x_{i}\right)$. To ensure an unbiased estimation of the estimated value $Z^{*}\left(V\right)$ to the true value Z(V), condition $E\left[Z^{*}\left(V\right)-Z\left(V\right)\right]=0$ is satisfied.

#### 2.2.3. Spatial Regression Model of Urban PM_2.5_ Socioeconomic Factors

The spatial difference of PM_2.5_ concentration in Chinese cities is significant and the influencing factors are diverse. This study discusses the influencing factors of PM_2.5_ from the perspective of socio-economic factors.

According to the existing research results, economic development, urbanization, and industrialization are the three main driving forces of urban development [21]. This study analyzes the effects of PM_2.5_ concentration changes from the following 10 factors, as shown in Table 1. Economic development includes economic growth and foreign investment. Urbanization includes population agglomeration, urban scale, urban greening, and transportation. Industrialization includes industrial structure, energy consumption, scientific and technological progress, and pollution emissions. The corresponding indicators of these 10 independent variables are per capita gross domestic product (GDP) (*X_1_*), foreign direct investment (FDI) amount (*X_2_*), population density (*X_3_*), urban built-up area (*X_4_*), urban greening rate (*X_5_*), number of owned vehicles (*X_6_*), industrial output value to GDP (*X_7_*), total energy consumption (*X_8_*), science and technology expenditure to GDP (*X_9_*), and soot emissions (*X_1_*_0_).

The above 10 indicators are included in the analysis model according to the traditional measurement model without considering the spatial effect. The model is as follows:

(3) $$\mathrm{lnY}=\beta_{0}+\beta_{1}lnX_{1}+\beta_{2}lnX_{2}+\beta_{3}lnX_{3}+\beta_{4}lnX_{4}+\beta_{5}X_{5}+\beta_{6}lnX_{6}\;+\beta_{7}X_{7}+\beta_{8}lnX_{8}+\beta_{9}X_{9}+\beta_{10}lnX_{10}+\varepsilon$$

In formula (3), *Y* is the PM_2.5_ concentration value. The values *X_1_,…,X_10_* are per capita GDP, FDI amount, population density, urban built-up area, urban greening rate, number of owned vehicles, industrial output value to GDP, total energy consumption, science and technology expenditure to GDP, and soot emissions, respectively. The value *β* is the model parameter. The value *ε* is a random error term. The natural logarithm of the independent variable is used to reduce the effect of heteroscedasticity on the model estimate. 

The change of PM_2.5_ concentration in each city is affected by surrounding cities and has spatial autocorrelation and spatial dependence. Therefore, this study extends the basic analysis model to the spatial econometric model. The calculation formula of the spatial lag model based on the basic analysis model is as follows:

(4) $$\mathrm{lnY}=\beta_{0}+\rho WY+\beta_{1}lnX_{1}+\beta_{2}lnX_{2}+\beta_{3}lnX_{3}+\beta_{4}lnX_{4}+\beta_{5}X_{5}\;+\beta_{6}lnX_{6}+\beta_{7}X_{7}+\beta_{8}lnX_{8}+\beta_{9}X_{9}+\beta_{10}lnX_{10}+\varepsilon$$

In the formula, *Y, X_1_, ..., X_10_*, *β*, and *ε* are defined as in formula (4). The value ρ is a spatial regression coefficient. The value *W* is a spatial weight matrix.

The calculation formula of the spatial error model based on the basic analysis model is as follows:

(5) $$\mathrm{lnY}=\beta_{0}+\beta_{1}lnX_{1}+\beta_{2}lnX_{2}+\beta_{3}lnX_{3}+\beta_{4}lnX_{4}+\beta_{5}X_{5}+\beta_{6}lnX_{6}\;+\beta_{7}X_{7}+\beta_{8}lnX_{8}+\beta_{9}X_{9}+\beta_{10}lnX_{10}+\varphi W\varepsilon +\mu$$

In the formula, *Y, X_1_, ..., X_10_*, *β*, and *ε* are defined as in formula (5). The value φ is the spatial error coefficient and μ is a random error vector of the normal distribution.

When choosing the spatial model, the ordinary least squares (OLS) method is used to estimate the spatial related constraint model and then the significance selection model of the Lagrange multiplier is compared.

If the Lagrange multiplier LM (lag) is more statistically significant than LM (error) and if R-LM (lag) is more significant than R-LM (error), the spatial lag model (SLM) is selected, otherwise structural equation modeling (SLM) is selected.

## 3. Results

### 3.1. PM_2.5_ Time–Dimension Evolution Characteristics 

#### 3.1.1. Annual Changes in PM_2.5_

**Figure 1:**
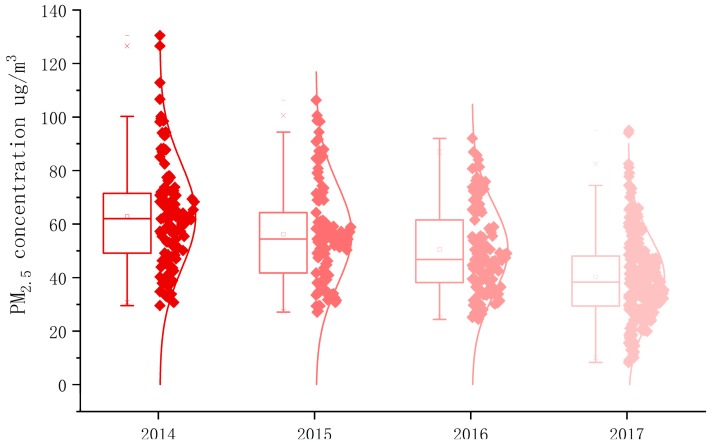
PM_2.5_ concentration value box line normal curve from 2014 to 2017.

The annual average value of PM_2.5_ concentration in Chinese cities from 2014 to 2017 showed a downward trend at an average of 62.9 μg/m^3^, 56.2 μg/m^3^, 50.1 μg/m^3^, and 40.3 μg/m^3^ for each year, respectively, as shown in Figure 1. The PM_2.5_ concentration value decreased by 22.6 μg/m^3^ in 2014–2017, a decrease of 35.93%. The drop in PM_2.5_ concentration was due to the initial success of cross-regional comprehensive governance in China in recent years. However, the annual average of PM_2.5_ concentration in 2017 was still 1.15 times higher than the secondary standard value (35 μg/m^3^). It was also higher than the PM_2.5_ concentration limit (10 μg/m^3^) set by the WHO and Western developed countries. 

By using K-S as normality test, the *p* values of PM_2.5_ in 2014–2017 were 0.001, 0.005, 0.003 and 0.019, which indicate that the concentration of PM_2.5_ was normally distributed every year. From the annual concentration distribution interval, the distribution frequency of the daily average PM_2.5_ concentration was close to the normal distribution. The normal curve of PM_2.5_ concentration decreased year by year and the peaks increased year by year. This result indicated that PM_2.5_ concentration in most cities decreased year by year and the pollution of fine particulate matter improved year by year. Cities with PM_2.5_ concentration between 35 and 70 μg/m^3^ had the greatest improvement, while those with mild and moderate pollution had smaller improvement. In 2014, PM_2.5_ concentration in some cities was still moderately high but, after 2017, the overall air quality of Chinese cities improved significantly.

#### 3.1.2. Seasonal and Monthly Changes of PM_2.5_

The change of PM_2.5_ concentration in China had a distinct seasonality, which was characterized by variation of high winter and low summer and middle spring and fall. In 2017, the average PM_2.5_ concentration in spring, summer, fall, and winter was 41.08 μg/m^3^, 26.17 μg/m^3^, 38.25 μg/m^3^, and 66.33 μg/m^3^, respectively. The difference in PM_2.5_ concentration between winter and summer was 40.16 μg/m^3^. This was related to the heating method of coal burning in winter in China, less rainfall, and sparse vegetation in winter. During the period from 2014 to 2017, the PM_2.5_ concentration in spring, summer, fall, and winter showed a downward trend year by year. The biggest PM_2.5_ drop was in summer; it fell from 44.08 μg/m^3^ in 2014 to 26.17 μg/m^3^ in 2017, a drop of 40.63%.

In terms of month-to-month changes, the monthly mean values of PM_2.5_ concentrations in 2014 and 2017 all showed U-shaped variation characteristics [3]. These values fell from January to May, were basically stable from June to September, while rising from October to December. The U-shaped inflection point appeared in August each year, that is, the PM_2.5_ concentration in August was the lowest in the whole year. In 2017, the average daily maximum and minimum values appeared in January (118.32 μg/m^3^) and August (17.12 μg/m^3^), respectively. Through smooth curve simulation, it was found that the U-type features had a flattening trend year by year and the PM_2.5_ concentration decreased the most in October, with a drop of 41.18%.

#### 3.1.3. Daily Changes of PM_2.5_

**Figure 2:**
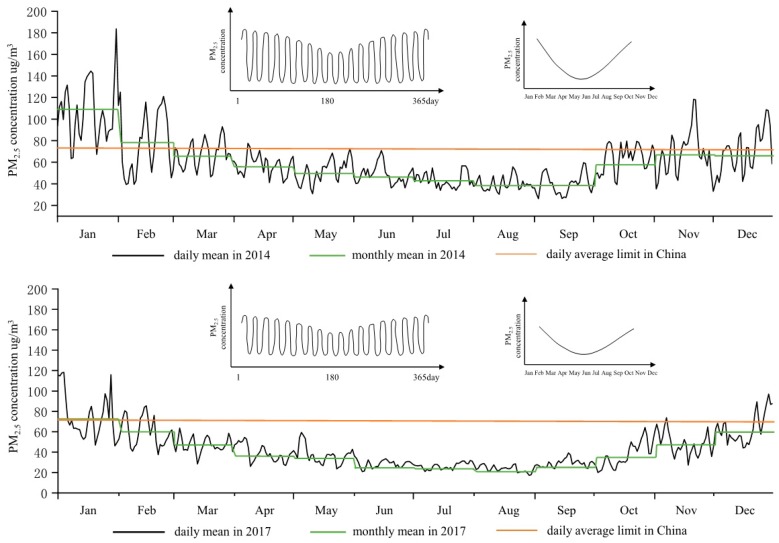
Daily and monthly mean values of PM_2.5_ concentration in 2014 and 2017.

The change of average daily concentration in Chinese cities presented a periodic U-pulse fluctuation rule [25], as shown in Figure 2. In spring and winter, the fluctuation cycle was short and the frequency was high, with a cycle of about 7 days. Summer and fall had long fluctuation periods and low frequency, with a period of about 10–15 days. From 2014 to 2017, the daily maximum and minimum PM_2.5_ concentrations showed a downward trend. The average daily maximum PM_2.5_ concentration decreased from 183.69 μg/m^3^ to 118.32 μg/m^3^, a decrease of 35.59%. The daily average minimum PM_2.5_ concentration decreased from 26.15 μg/m^3^ to 17.12 μg/m^3^, a decrease of 34.53%. The range of maximum and minimum values dropped from 157.54 μg/m^3^ in 2014 to 101.20 μg/m^3^ in 2017. The result showed that the urban air quality in China was getting better year by year, but the average daily PM_2.5_ concentration in winter and spring was still high. 

The average daily PM_2.5_ concentration in Chinese cities reached 93.42% in 2017. The proportion of mild pollution and moderate pollution was 5.48% and 1.10%, respectively. In 2014, the compliance rate was only 77.81%. In terms of the annual variation of over-standard, the over-standard days in Chinese cities in the years 2014–2017 were 81 days, 60 days, 50 days, and 24 days, respectively. The number of days exceeding the standard decreased year by year and the rate of exceeding the standard decreased from 22.19% to 6.58%. 2017 was the year with the largest number of days reduced, with 26 days less than the previous year.

#### 3.1.4. Hourly Changes of PM_2.5_

**Figure 3:**
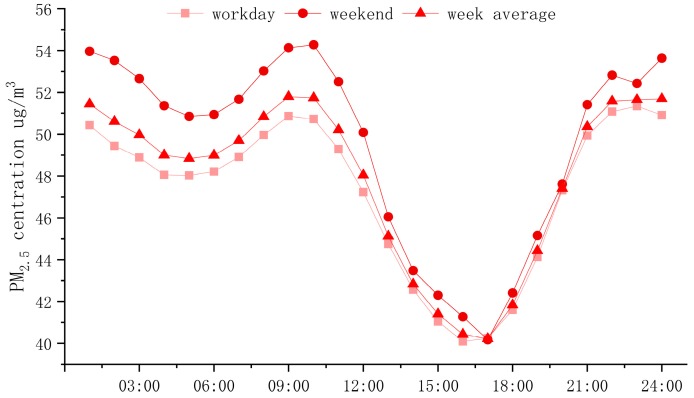
Daily variation trend of PM_2.5_ concentration in 2017.

China’s urban PM_2.5_ concentration showed a bimodal distribution with hourly curve in 2017 (Figure 3). The daily average peak value of PM_2.5_ concentration was 51.79 μg/m^3^ at 10:00 and the valley value was 40.22 μg/m^3^ at 16:00, with a difference of 11.57 μg/m^3^. 

Traffic peak was concentrated in the morning, when the amount of particulate matter discharged from urban traffic increased. Moreover, the atmosphere mixed layer was low in height and the inversion layer was likely to appear close to the ground, leading to adverse diffusion of pollutants and forming the first peak of the PM_2.5_ concentration. In the afternoon, traffic pollution emissions were lower and the convective movement in the atmosphere was enhanced, which was conducive to the spread of pollutants; thus, a trough was formed at around 16:00. After the evening, traffic pollution during rush-hour traffic and cooking fume pollution continued to increase and the PM_2.5_ concentration gradually increased. Moreover, the low valley electricity price caused industrial production to increase, which led to the increase of PM_2.5_ pollution. As a result, the second PM_2.5_ concentration peak occurred during the night to the early morning hours.

There was no “weekend effect” in PM_2.5_ concentration in Chinese cities. Conversely, the PM_2.5_ concentration on weekends was higher than on working days. This may be because the 338 cities in this study contain more small-sized cities and people work and live in a smaller radius. Industrial production activities in small cities have high weekend operating rates and emit more pollutants into the atmosphere [34]. This shows that the changes in people’s daily life and rest over the weekend have no significant impact on PM_2.5_ pollution.

### 3.2. PM_2.5_ Spatial Dimension Evolution Characteristics

#### 3.2.1. Evolution of PM_2.5_ Spatial Pattern

The geostatistics are explored by using the semivariance function to explore the spatial variability and correlation of PM_2.5_ concentration [3]. The fitting parameters are shown in Table 2. The result show that the nugget variance coefficient is greater than 0.726, which indicates that PM_2.5_ concentration has a certain spatial autocorrelation. The range of PM_2.5_ concentration is 71.53 km, which is larger than the average distance of monitoring point. The semivariance function fitting determination coefficient *R*^2^ is 0.863, greater than 0.5, showing a significant data fitting effect. The result supports using Kriging spatial interpolation analysis. Therefore, based on the analysis of spatial variability characteristics, the values of PM_2.5_ concentration in Chinese cities are estimated by the ordinary Kriging interpolation method and the spatial distribution characteristics of PM_2.5_ concentration in Chinese cities are analyzed.

**Figure 4:**
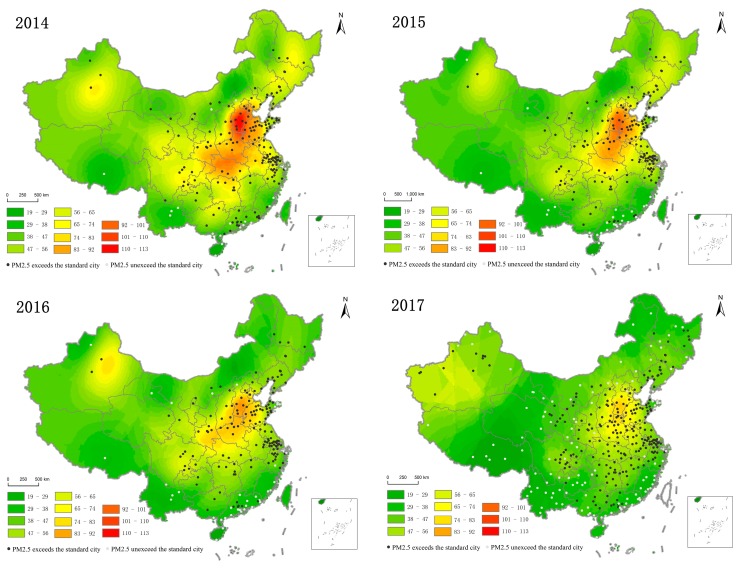
Spatial distribution of annual average PM_2.5_ concentration in Chinese cities in 2014–2017.

China’s PM_2.5_ concentration high and low value east–west boundary line is the Hu Huanyong Line [35], while the north–south boundary line is the Yangtze River. The intersection area east of the Hu Huanyong Line and north of the Yangtze River was a cluster of high-polluting urban clusters, including Beijing, Hebei, Shandong, Henan, and Hubei, as shown in Figure 4. The range covers moderately and heavily polluted cities with an annual average of 81 μg/m^3^ or more. The areas heavily polluted by PM_2.5_ were concentrated in the contiguous zone and the outward diffusion gradually became better. There was a certain coupling between PM_2.5_ concentration and socio-economic activity of the region. The east of the Hu Huanyong Line had a developed economy, dense population, and concentrated industrial layout, resulting in serious PM_2.5_ pollution in the east. The geomorphological area of the North China Plain made PM_2.5_ pollution conducive to diffusion and mutual influence, resulting in increased pollution. The average annual PM_2.5_ concentration to the west of the Hu Huanyong Line (except the central part of Xinjiang) and the south of the Yangtze River is low, including Tibet, Yunnan in the southwestern regions, and the southeast coastal regions. Furthermore, cities with an average annual PM_2.5_ concentration reaching the secondary standard limit were mainly distributed in this region.

From the perspective of spatial evolution in 2014–2017, the scope of PM_2.5_ pollution gradually narrowed and the pollution situation significantly improved. The pollution range in the Beijing–Tianjin–Hebei region shrank toward the southwest, and the pollution ranges in Henan and Shandong shrank to the north and west, respectively. This is mainly because the local government increased supervision and prevention and controlled measures for regions with heavy pollution, which reduced the local PM_2.5_ concentration and the air pollution range gradually spread to cities with poor supervision.

#### 3.2.2. Spatial Distribution of PM_2.5_ Annual Average Exceeding the Standard

According to China’s environmental air quality standard (GB 3095-2012), the PM_2.5_ concentration in most cities exceeded the standard to different degrees. In 2014–2017, Chinese PM_2.5_ concentration exceeded the standard range by more than two-thirds of the total area, forming the pollution pattern of high value in the central and eastern China and low value in the peripheral areas (Figure 4).

In 2014, a total of 190 cities were included in the monitoring but, of them, only 18 cities in the coastal and inland regions did not exceed the standard. Other cities had different levels of exceeding the standard, with the proportion exceeding the standard as high as 90.53%. By 2015, the scope of the over-standard had been reduced to 83.16% of the monitored cities. The annual average PM_2.5_ concentration in 91.05% of cities decreased to different degrees, with an average decline of 8.08 μg/m^3^. Among them, Shijiazhuang City, the capital of Hebei Province, where pollution was serious, had the biggest drop, as high as 34.90 μg/m^3^. In 2016, the average annual value of PM_2.5_ concentration in most cities fell below 65 μg/m^3^ and the average decline was reduced to 6.36 μg/m^3^, which was slower than the decline in 2015. The proportion of cities exceeding the standard decreased to 80.26%. The biggest drop was 19.10 μg/m^3^ for Langfang City of Hebei Province. In 2017, the annual average value of PM_2.5_ concentration in the area above 65 μg/m^3^ shrank, with an average decrease of 6.87 μg/m^3^. China’s urban air quality continues to improve, with the number of cities exceeding the standard steadily decreasing. Hebei Province was still the most polluted area in China and a key area for PM_2.5_ emission reduction and air pollution prevention.

During 2014–2017, the proportion of cities that met the PM_2.5_ concentration standard rose from 9.7% to 33.88%. The compliance area slowly expanded from the periphery to the middle and the air quality gradually improved.

#### 3.2.3. Spatial Distribution of the Daily Average of PM_2.5_ Exceeding the Standard Number

**Figure 5:**
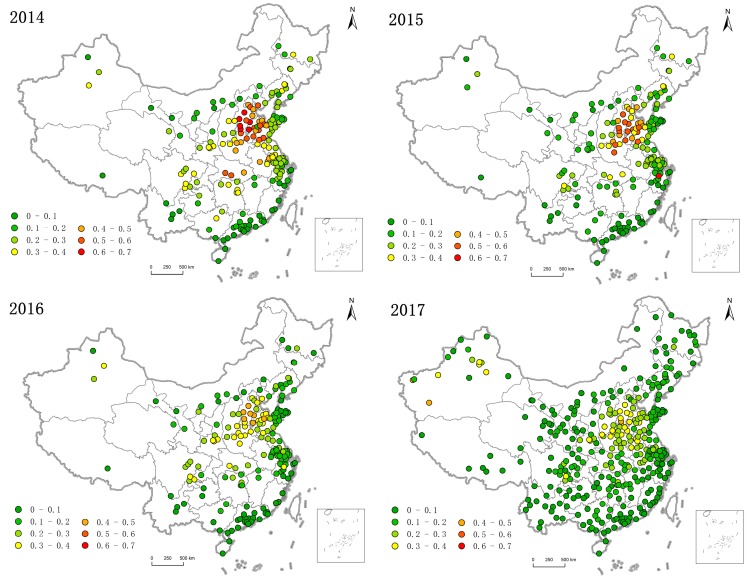
Proportion of days when daily average PM_2.5_ concentration exceeds the standard in 2014–2017.

Referring to the Ambient Air Quality Index (AQI) Technical Regulations (HJ633-2012), this study calculated the proportion of days with excessive daily mean according to the second-level daily mean standard (75 μg/m^3^). The proportion of over-standard days in China’s urban daily average showed a similar spatial distribution pattern to the annual average over-standard ratio. The serious areas exceeding the standard were mainly concentrated in Hebei, Henan, and Shandong, with the spatial pattern gradually spreading out from the pollution core area (Figure 5).

The proportion of over-standard days in Chinese cities in 2014 ranged from 0.91% to 68.49%, with an average over-standard ratio of 26.25%. However, in 2017, the proportion of over-standard days in all cities ranged from 0.18% to 48.09%, the average over-standard ratio fell to 12.78%, and the over-standard rate showed a downward trend.

From the perspective of specific cities, in 2014, pollution was the most serious in Xingtai, Baoding, and Handan cities in Hebei Province, exceeding 64%, as measured by the proportion of over-standard days. By 2017, the proportion of over-standard days in these three cities had dropped to 36%, but it was still relatively serious compared to other cities. Areas with high pollution were most clearly reduced and the proportion of days exceeding the standard in Hebei, Henan, Shandong, Shanxi, and Jiangsu provinces was significantly reduced. This result showed that the regional integrated air pollution control implemented by the Chinese government in recent years has achieved remarkable results [36].

#### 3.2.4. PM_2.5_ Spatial Agglomeration Analysis

**Figure 6:**
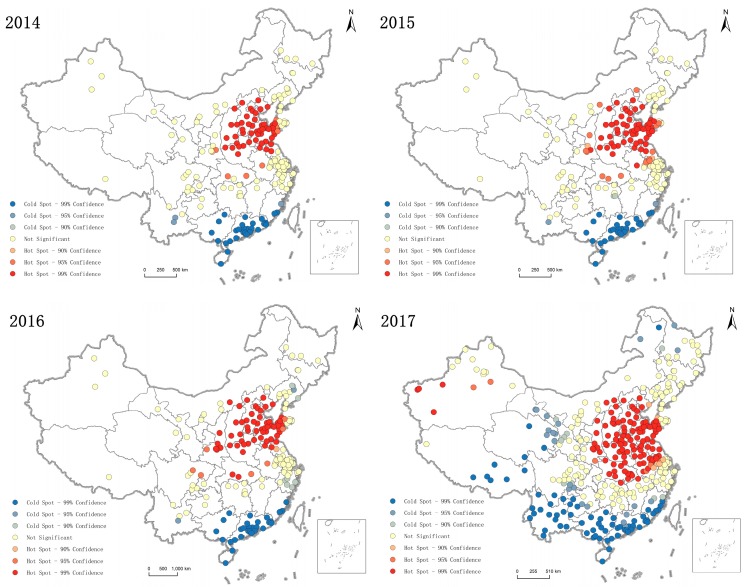
Spatial distribution of cold and hot spots with PM_2.5_ concentration during 2014–2017.

By calculating the Moran’s I, we explored the spatial agglomeration of the annual average PM_2.5_ concentration in Chinese cities and identified high and low pollution hotspots. The results showed that Moran’s I was 0.291, 0.327, 0.329, and 0.504 for each year in 2014–2017, respectively, which all passed the significance test of 1%. This finding showed that the annual average of PM_2.5_ concentration in Chinese cities had a high spatial positive correlation and cities with similar pollution levels tended to have spatial clustering distribution, as shown in Figure 6.

PM_2.5_ concentration hot spots in 2014 were mainly concentrated in Hebei Province, Shandong Province, Shanxi Province, Henan Province, Beijing City, and Tianjin City. In this region, high PM_2.5_ concentration appeared to form a stable and continuous contaminated contig and the air quality was poor. By 2017, the PM_2.5_ concentration hotspots had expanded to the south and the area gradually expanded. Hubei Province, Jiangsu Province, and Anhui Province became hot spots, as did Urumqi City and Kezhou City in Xinjiang Province. There was a large desert in the west of Xinjiang Province and dusty weather in the dusty areas was frequent. The PM_2.5_ concentration over the desert area would be greater than the particle concentration in the vegetation covered area [19].

In 2014, PM_2.5_ concentration cold spots were mainly concentrated in Guangdong, Fujian, and Guangxi provinces on the southeast coast [28], which was related to the strong coastal wind and conducive to diffusion [3]. By 2017, as the number of PM_2.5_ monitoring points increased, the area of cold spots also gradually expanded, showing a trend of moving northward and westward. The underdeveloped inland regions of Tibet, Yunnan, Qinghai, and Gansu provinces had also become cold spots. The cold spots became a continuously stable area with good air quality. The middle and upper reaches of the Yangtze River form frequent alternating zones of polluted air and fine air. The air quality in southern China has improved significantly.

### 3.3. Socio-Economic Factors Affecting PM_2.5_ Concentration

Based on the above Equation (3), the socio-economic factors influencing the distribution of PM_2.5_ concentration were analyzed by using the PM_2.5_ density and the socio-economic data of Chinese cities. There was spatial correlation between PM_2.5_ concentration and independent variables, and thus, spatial effects were considered in the model building. The correlation between the independent variables was analyzed in SPSS (IBM SPSS, Somers, NY, USA), and the correlation coefficients were all less than 0.5. By using OLS to estimate the model, the variance expansion factor of each variable was obtained, which was less than the critical value 10, indicating that the model did not have a multicollinearity problem. Using OLS estimation to consider the spatial correlation constraint model, it was found that LMLAG was statistically more significant than LMERR, R-LMAG was significant, and R-LMERR was not significant. Therefore, the SLM was selected for analysis. The model estimation results are shown in Table 3. Comparing the results of the OLS and SLM models, the fitting degree *R*^2^ in the OLS estimation was 0.406 and the *R*^2^ in the spatial lag model estimation was 0.626. It can be seen that the fitting degree of the model was significantly improved after considering the spatial correlation.

From the perspective of economic development, urbanization, and industrialization, this study evaluated the socio-economic driving force of PM_2.5_ concentration in Chinese cities. In the long run, economic growth, industrialization, and urbanization were important driving forces of PM_2.5_ pollution in Chinese cities, which was consistent with some earlier studies [21]. The high PM_2.5_ concentration was mainly concentrated in metropolitan areas with large population, high GDP, and a large proportion of urbanization and industrialization [19]. Human activities were often the source of PM_2.5_ concentration.

The influencing factors of the model include economic development, population agglomeration, industrial structure, energy consumption, foreign investment, urban scale, urban greening, transportation, technological progress, and pollution emissions. Among them, the total energy consumption, urban built-up area, urban greening rate, and PM_2.5_ concentration were not significant, indicating that these three had no significant impact on PM_2.5_ concentration changes in China.

Population agglomeration, industrial structure, foreign investment, transportation, and pollution emissions were important factors to promote PM_2.5_ pollution. Population density, industrial output value to GDP, FDI amount, number of owned vehicles, and soot emissions were significantly positively correlated with urban PM_2.5_ concentrations. For a 1% increase in each of these five factors, PM_2.5_ concentration increased by 0.107%, 0.010%, 0.023%, 0.096%, and 0.040%. Among them, population density contributed the most to the change of PM_2.5_ concentration (*r* = 0.107, *p* < 0.01) and the production and living of urban population agglomeration directly aggravated PM_2.5_ pollution [37]. The number of owned vehicles (*r* = 0.096, *p* < 0.05) also had a large impact, indicating that the rapid growth of vehicle ownership and the increase of vehicle exhaust emissions were one of the important causes of PM_2.5_ pollution. Air pollution prevention and control should increase the treatment of automobile emissions. From the perspective of the transportation process, local transportation and regional transportation have significantly promoted the PM_2.5_ pollution load in the region [38]. The FDI amount (*r* = 0.023, *p* < 0.1) had a significant positive impact on PM_2.5_ concentration. This showed that FDI inflow contributed to air pollution and the “pollution shelter” hypothesis was established in China [26]. When FDI was selected for location, it was affected by environmental regulations and environmental governance costs. Moreover, China has become a refuge for highly polluting foreign-funded enterprises. Industrial output value to GDP (*r* = 0.010, *p* < 0.01) was significantly positively correlated with PM_2.5_ concentration. This showed that China was still in the period of industrial development and industrial agglomeration was the main source of environmental pollution. At the same time, industrial activities emitted a large amount of pollutants, such as smoke and dust (*r* = 0.096, *p* < 0.01), which was an important factor causing PM_2.5_ pollution. Heavy pollution areas in Shandong, Henan, Hebei, Shanxi, and other provinces were the most important coal and steel industrial agglomerations in China and the spatial agglomeration was one of the important factors leading to PM_2.5_ pollution.

Economic growth and technological progress have significantly promoted the improvement of urban PM_2.5_ concentration. Per capita GDP had a significant negative impact on PM_2.5_ concentration, for every 1% increase in per capita GDP, PM_2.5_ concentration fell by 0.263%. There was an inverted U-shaped environmental Kuznets curve (EKC) between per capita GDP and PM_2.5_ concentration, which is consistent with previous studies [39]. China has crossed the inflection point of the inverted U-shaped curve [40]. Economic development has promoted an improved atmospheric environment. China’s economic growth has passed the stage of sacrificing environmental quality. In addition, the proportion of science and technology expenditure to GDP was significantly negatively correlated with PM_2.5_ concentration. For every 1 percentage point increase in the proportion of science and technology expenditure, the PM_2.5_ concentration decreased by 0.040%. Scientific and technological progress could prevent and control air pollution, and the Porter hypothesis was verified for China’s air pollution control. Appropriate environmental regulation stimulated technological innovation of enterprises. Enterprises reduce pollution emissions and achieve a win–win situation while reducing costs and improving product competitiveness.

## 4. Discussion

In the past few years, haze caused by high PM_2.5_ concentration has been of increasing concern to the Chinese public and government. China faced a series of air pollution threats in the process of rapid urbanization and industrialization. The phenomenon of haze is not unique to China. Britain, the United States, Germany, France, and other developed countries have also experienced intensive and large-scale haze in their processes of industrialization. However, these countries cured air pollution through industrial upgrading and relocation and various government initiatives. In a review of air environment governance practices and experiences in different countries, the Chinese government has issued several laws, regulations, and policies on atmospheric prevention since 2013. Since then, there have been remarkable achievements in air pollution mitigation and haze control in China.

In recent years, the Chinese government has adopted a multi-level, cross-regional, and multi-directional control model for haze pollution in key areas. The model consists of a hierarchical vertical linkage structure consisting of a country-urban agglomeration-city. This model cooperated with the trans-district and transverse linkage governance mode of several provinces, municipalities, and administrative regions. The linkage mechanism included industry access, energy structure, green transportation, cross-regional assistance, monitoring and early warning, and consultation and accountability. In terms of specific control measures, over the past 5 years, China has steadily intensified efforts to control haze pollution. These measures included the treatment of “scattered pollution” enterprises, the implementation of ultra-low emission transformation of coal-fired thermal power units, the elimination of yellow-standard cars and old cars, the elimination of small coal-fired boilers, and the emergency response of heavily polluted weather, etc. The Chinese government has formed a coordinated regional management model for haze pollution, formed based on regional linkage and the participation of government-led, enterprise-oriented, and public and social organizations. This model has become the fundamental path and inevitable choice to win the battle for blue skies.

The driving factors of PM_2.5_ concentration change in China are complex [41]. The anthropogenic factors were the most important driving factors affecting PM_2.5_ concentration, including industrial pollution, coal combustion, motor vehicle emissions, dust, biomass combustion, and garbage incineration [31]. In addition, natural factors, such as atmospheric circulation [42], topography, extreme weather, and regional transmission, also had great influences on PM_2.5_ concentration. Due to the large spatial inequality of industrial structure, energy structure, and physical and geographical conditions, the key factors driving the change of PM_2.5_ varied across different regions in China. Atmospheric pollutants have complexity and long-term treatment and the impact of urban economic growth and urbanization expansion on air pollution in China is worth exploring. The public cries on air pollution and the crisis of different environmental types are also the future focus, needing in-depth exploration. Finally, it is also important to explore empirical studies on the impact of PM_2.5_ or other air pollutants on public health, which can expand our understanding of the disastrous consequences of the air quality degradation in China [28].

## 5. Conclusions

Previous studies have paid attention to the changing pattern of PM_2.5_ concentration and its socio-economic determinants [37]. Most studies have focused on regional PM_2.5_ concentration, but have seldom explored the national geographic variation. However, the research at the large territorial scale is critical to understand the socio-economic mechanism of air pollution. Therefore, this study advanced our understanding of the spatial heterogeneity of pollution and the dynamic relationship between economic development gradients and pollution concentration levels. The multi temporary (i.e., daily, monthly, and yearly) data of PM_2.5_ concentration was more closely related to the public’s ordinal life and exposure to pollutants, which is more practical and instructive for spatial policy implications for the country’s environment governance. Moreover, the longitudinal data can better reflect the policy effectiveness of the Chinese government in reducing PM_2.5_ concentration and corresponding characteristics of spatial changes. From the data aspect, the PM_2.5_ ground monitoring data was much more reliable and accurate than the remote sensing inversion data. Based on the above research, the following conclusions were mainly drawn:

(1) The time dimension change of PM_2.5_ concentration had the following characteristics. First, from the perspective of annual changes, the annual average of PM_2.5_ in Chinese cities in 2014–2017 dropped year by year, from 62.9 μg/m^3^ to 40.3 μg /m^3^, and urban air quality improved year by year. Second, from the perspective of monthly changes, the monthly average of PM_2.5_ showed the characteristics of a U-type fluctuation and change. Along with with high winter and low summer, spring and fall were transitional periods, and the U-type characteristics were flattened year by year. Third, from the perspective of day-by-day data, the average daily concentration changes of Chinese cities presented the periodic u-pulse fluctuation rule. The spring and winter fluctuation periods were short and the frequency was high. The summer and fall fluctuation periods were long and the frequency was low. Finally, from the perspective of time-to-time changes, the PM_2.5_ hourly curve showed a bimodal distribution and the peak was formed after the morning and evening travel peak.
(2) The PM_2.5_ spatial dimension had the following characteristics. The PM_2.5_ concentration was low in the east and high to the west of the Hu Huanyong Line. Similarly, it was high in the north and low to the south of the Yangtze River. The intersection area east of the Hu Huanyong Line and north of the Yangtze River was a cluster of highly polluted cities, including Beijing, Hebei, Shandong, Henan, and Hubei. These areas contained moderately and heavily polluted cities, with an annual average of 81 μg/m^3^ or more. Rapid economic and social development of urban agglomeration was the main determinant of PM_2.5_ pollution.
(3) In Chinese cities, PM_2.5_ exceeded the limit by more than two-thirds of the total area over the years, presenting a pollution pattern of high in the middle and low on the four sides. However, the over-standard ranged to the middle every year and the air pollution situation improved.
(4) The annual average value of PM_2.5_ in Chinese cities had obvious spatial agglomeration and formed hot spots and cold spots in dynamic changes. Hot spots were mainly concentrated in Hebei Province, Shandong Province, and Beijing City, among others. The high concentration of PM_2.5_ concentration formed a stable and continuous pollution contiguous zone. The cold spot areas were mainly concentrated in the southeast coastal areas, which were related to the diffusion of coastal wind.
(5) PM_2.5_ pollution had spatial autocorrelation and the model considering spatial effects was superior to the ordinary model. Economic growth, industrialization, and urbanization increased PM_2.5_ concentration in Chinese cities. Population agglomeration, industrialization, foreign investment, transportation, and pollution emissions are important factors to promote PM_2.5_ pollution. Among them, urban population density contributed most to PM_2.5_ concentration. The “pollution shelter” hypothesis was established for China, which remains a refuge for highly polluting foreign-funded enterprises. Economic development and scientific and technological progress significantly promoted the improvement of urban PM_2.5_ concentration. There was an inverted U-shaped environmental Kuznets curve (EKC) between per capita GDP and PM_2.5_ concentration. China has crossed the inflection point of the inverted U-shaped curve.

## Figures and Tables

**Table 1:** Social and economic factors affecting PM_2.5_ concentration in Chinese cities.

Drivers	Factors	Independent Variable	Number
Economic development	Economic growth	Per capita GDP [25]	*X*_1_
	Foreign investment	Foreign direct investment amount [26]	*X*_2_
Urbanization	Population agglomeration	Population density [27]	*X*_3_
	Urban scale	Urban built-up area [28]	*X*_4_
	Urban greening	Urban greening rate [29]	*X*_5_
	Transportation	Number of owned vehicles [30]	*X*_6_
Industrialization	Industrial structure	Industrial output value to GDP [31]	*X*_7_
	Energy consumption	Total energy consumption [32]	*X*_8_
	Scientific and technological progress	Science and technology expenditure to GDP	*X*_9_
	Pollution emissions	Soot emissions [33]	*X*_10_

Note: GDP: Gross Domestic Product.

**Table 2:** Theoretic model and parameters of semivariance of PM_2.5_ concentration in Chinese cities.

Variable	Nugget Variance	Structural Variance	Proportion	Range (km)	Residual Square RSS	Coefficient of Determination *R*^2^	Theoretical Model
PM_2.5_	0.327	0.451	0.726	71.534	3.14 × 10^−4^	0.863	Gaussian

**Table 3:** Result of the models.

Drivers	Influencing Factor	Independent Variable	OLS		SLM	
			*r*	*p*	*r*	*p*
		Constant	4.41441 ***	0.00000	0.87407 *	0.09802
Economic development	Economic growth	Per capita GDP	−0.27561***	0.00059	−0.26298 ***	0.00001
	Foreign investment	Foreign direct investment amount	0.06319 ***	0.00056	0.02288 *	0.09638
Urbanization	Population agglomeration	Population density	0.13741 ***	0.00030	0.10663 ***	0.00018
	Urban scale	Urban built-up area	−0.00759	0.90826	−0.00806	0.87284
	Urban greening	Urban greening rate	0.00123	0.79717	−0.00119	0.74338
	Transportation	Number of owned vehicles	0.11840 **	0.02025	0.09557 **	0.01340
Industrialization	Industrial structure	Industrial output value to GDP	0.00939 ***	0.00191	0.00986 ***	0.00002
	Energy consumption	Total energy consumption	−0.02118	0.64834	0.03408	0.33715
	Technological progress	Science and technology Expenditure to GDP	−0.08875 ***	0.00549	−0.04031 *	0.09514
	Pollution emissions	Soot emissions	0.09570 ***	0.00002	0.03963 **	0.01682
		*R* ^2^	0.40629 ***	0.00005	0.62593 ***	0.00004
		LMLAG	100.38681 ***	0.00000		
		LMERR	54.92353 ***	0.00000		
		R-LMLAG	47.70872 ***	0.00000		
		R-LMERR	2.24540	0.13401		

Note: *, **, and *** mean significant at 10%, 5%, and 1% level, respectively. OLS: ordinary least squares; SLM: spatial lag model; LMLAG: Lagrange Multiplier (lag); LMERR: Lagrange Multiplier (error); R-LMLAG: Robust of Lagrange Multiplier (lag); R-LMERR: Robust of Lagrange Multiplier (error).
